# Muscle Contractile Properties Measured by the Tensiomyography (TMG) Method in Top-Level Football Players of Different Playing Positions: The Case of Serbian Super League

**DOI:** 10.3390/ijerph20020924

**Published:** 2023-01-04

**Authors:** Lazar Pajović, Lazar Toskić, Veroljub Stanković, Ljubiša Lilić, Borislav Cicović

**Affiliations:** 1 Faculty of Physical Education and Sport, University of East Sarajevo, 71420 Pale, Bosnia and Herzegovina; 2 Faculty of Sport and Physical Education, University of Priština–Kosovska Mitrovica, 38218 Leposavić, Serbia; 3 Faculty of Sport, University “Union–Nikola Tesla”, 11070 Belgrade, Serbia

**Keywords:** tensiomyography, muscle contractile properties, football, defenders, midfielders, forwards

## Abstract

The aim of this study is to investigate the differences in muscle contractile properties measured by the TMG method between top-level football players of different playing positions. The sample consisted of 57 football players from the Serbian Super League, divided into three groups: defenders—DF, midfielders—MF, and forwards—FW. Muscles included in the study were the Rectus Femoris (RF), Vastus Medialis (VM), Vastus Lateralis (VL), Biceps Femoris (BF), and Semitendinosus (ST) of the right (R) and left (L) leg. The TMG parameters used in this study were contraction time (Tc), delay time (Td), relaxation time (Tr), maximal displacement (Dm), and sustain time (Ts). The ANOVA results showed that differences in TMG parameters between top-level football players of different playing positions are small and exist only in the Tr of RF (F = 4.658, *p* = 0.014), BF (F = 4.433, *p* = 0.016), and ST muscle (F = 3.808, *p* = 0.028), and the Tc (F = 3.214, *p* = 0.048) and Td (F = 3.705, *p* = 0.031) of the VM muscle. All differences were detected between DF and FW players, and all differences were in the left (non-dominant) leg. The results obtained in this study indicate that the training and selection process in football, from the aspect of functional and mechanical muscle properties, should be similar for all players, regardless of playing position. It has been shown that TMG has relatively low sensitive strength for detecting differences between football players of different playing positions.

## 1. Introduction

Football belongs to the group of team sports that can be classified as complex sports. The nature of its complexity can be perceived from the biomechanical aspect (movements around all axis and through all planes, different levels of strength and power manifestation, etc.), the energetic aspect (high and low exercise intensities), technical skills, tactical preparedness, etc. [1,2]. In addition, the football team is composed of eleven players, divided into different playing positions, where each position has its own tasks and psychophysical demands. Understanding the nature of players’ position characteristics in team sports, such as football, can be of great value for sports training, the selection process, and the achievement of top-level results.

So far, numerous studies have investigated differences in various characteristics between football players of different playing positions. Previous studies investigated differences between football players of different playing positions in anthropometric characteristics [3,4,5,6,7], physiological profile [3,6,8,9,10], motoric abilities [4,9,10,11], muscle strength and power [12,13,14], etc. These studies have shown that there are significant differences in numerous measured parameters between football players of different playing positions.

However, very few studies have investigated the differences between football players of different playing positions in muscle contractile properties measured by the method of tensiomyography (TMG) [15,16,17]. Tensiomyography is one of the methods that is being frequently used of late to assess muscle functional and mechanical properties; it is a non-invasive method developed to assess muscle properties in response to electrical stimuli [18,19,20]. This method provides information about muscle stiffness, contraction velocity, and, indirectly, the type of predominant skeletal muscle fibers, muscle fatigue, body asymmetry, etc. [15,18,21]. Besides other sports, TMG is widely used in football [15,16,17,22,23,24,25,26,27,28,29,30,31].

As previously emphasized, to the best of the author’s knowledge, only three studies were previously conducted that dealt with muscle contractile parameters measured by the TMG method in football players of different playing positions. Two of these studies have focused on Spanish male football players [15,17], while the third study investigated inter-limb differences in female football players [16]. Muscle contractile properties are important characteristics of football players that allows them to perform sprinting, jumping, tackling, and kicking [32,33]. Following the aforementioned deficiency of studies on this topic and the importance of muscle contractile properties in football, there is a need for further investigation of football players’ characteristics according to their position in the team from the aspect of muscle contractile properties measured by the TMG method. Accordingly, the aim of this study is to investigate the differences in muscle contractile properties measured by the TMG method between top-level football players of different playing positions. The alternative hypothesis of the research considered by the authors is that there will be significant differences in measured parameters between groups. The results of this study could give us insight into specific characteristics of playing positions in football regarding muscle functional and mechanical properties, which could contribute to the development of the training and selection process in football.

## 2. Materials and Methods

### 2.1. Subjects

The sample participants consisted of 57 football players divided into three groups: defenders—DF (N = 22, Age = 24.09 ± 5.1 years, BW = 82.09 ± 7.07 kg, BH = 186.4 ± 5.6 cm), midfielders—MF (N = 15, Age = 26.8 ± 3.7 years, BW = 79.06 ± 7.2 kg, BH = 182.2 ± 6.5 cm), and forwards—FW (N = 20, Age = 25.5 ± 5.7 years, BW = 79.6 ± 5.2 kg, BH = 183.5 ± 5.2 cm). The players were members of teams from the Serbian Super League; Radnički Niš (N = 17), Radnički Kragujevac (N = 18), and Novi Pazar (N = 22). Subjects included in the study were professional athletes, with a minimum of 5 years of experience in senior football professional activity (inclusion criteria). All participants were healthy and did not participate in any physical activity 12 h prior to the measurement (exclusion criteria). Before measurements, the study purpose and the protocol were explained to each participant, and consent for participation in the study was obtained. The study was conducted in accordance with the ethical standards of the Declaration of Helsinki and the rules of the Ethics Committee of the Faculty of Physical Education and Sport, University of East Sarajevo (protocol code 1628/22).

### 2.2. Procedures

Muscles included in the study were knee extensor and flexor muscles Rectus Femoris (RF), Vastus Medialis (VM), Vastus Lateralis (VL), Biceps Femoris (BF), and Semitendinosus (ST) of the right (R) and left (L) leg. The standardized protocol for TMG measurements was used [34,35,36]. For measurements of RF, VM, and VL muscles, all subjects lay on their back on a massage table, the knee was supported with a support pillow, angle in the knee was 120° angles. For measurements of BF and ST muscles, participants lay face down on a bed and their legs were supported with a support pillow [34,35,36].

**Figure 1:**
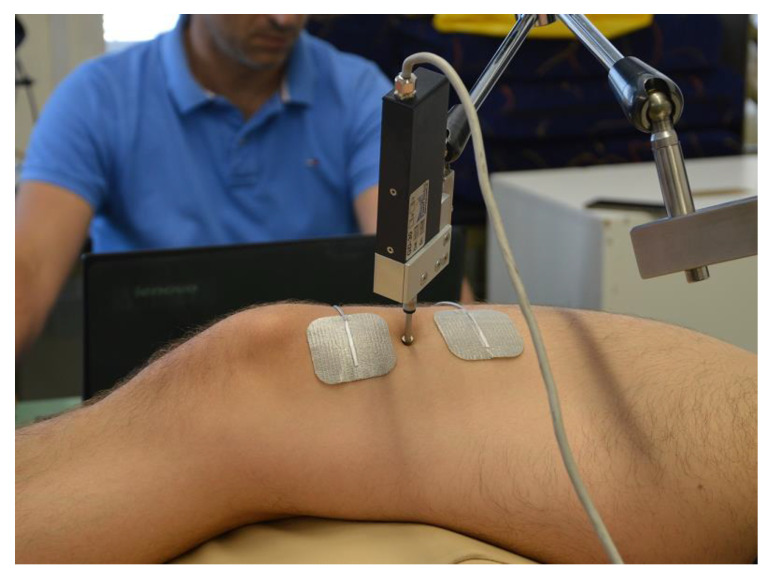
TMG measurement on muscle Vastus Medialis.

Measurements were taken under isometric conditions. Electrodes (Pals Platinum, model 895220 with a multi-stick gel, Axelgaard Manufacturing Co., Ltd., Fallbrook, CA, USA) were positioned inside muscle borders (proximally and distally) and the sensor (GK40, Panoptik, Ljubljana, Slovenia) location was at the midpoint between both electrodes, at the center of the muscle belly (Figure 1) [37]. After positioning the electrodes, electrical stimuli (TMG 100—TMG-BMC d.o.o., Ljubljana, Slovenia) was given (10 mA) and repeated at 5 s intervals [21]. The current increase was 20 mA until reaching the supramaximal muscle response (90–110 mA) [38]. The total duration of the procedure for both legs in a single subject was between 8 and 12 min. No discomfort was complained of by the subjects.

Measurements were conducted from 09:00 A.M. to 17:00 P.M. All measurements were done at the end of the season (June 2022), at the club’s facilities, and were performed by the experienced staff. The tensiomyography parameters used in this study were contraction time (Tc), delay time (Td), relaxation time (Tr), maximal displacement (Dm), and sustain time (Ts) [35].

All results of measurements were collected in special TMG software 3.6 (TMG-BMC d.o.o., Ljubljana, Slovenia). For every subject, data was separately analyzed on the dominant and non-dominant leg muscles. For all measured parameters, the best value was saved and analyzed (the smallest value of Tc and the highest value of Dm).

### 2.3. Statistical Analyses

The statistical procedures used in the study included descriptive statistics (Mean, SD), and univariate analyses of variance (ANOVA), while the distribution of normality was calculated by the Kolmogorov-Smirnov (KS) test. Univariate analyses of variance (ANOVA), along with the Bonferroni post hoc test, were applied to determine differences between football players of different playing positions in measured TMG parameters. The level of statistical significance was set at 95% with *p* < 0.05 [39]. All the statistical procedures were performed in the SPSS19 (IBM) program, IBM Armonk, New York, NY, USA.

## 3. Results

**Figure 2:**
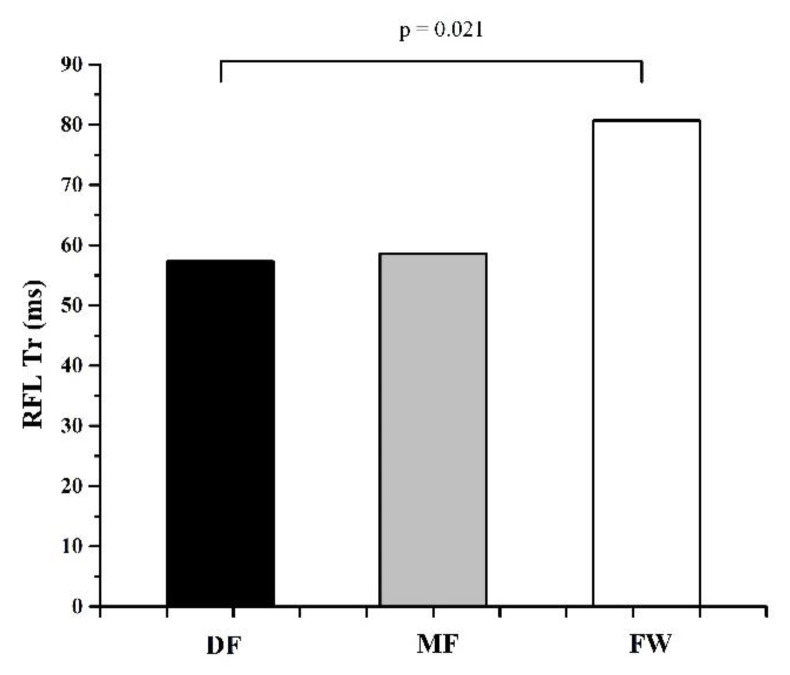
Differences between football players of different playing positions in Tr of RFL muscle.

**Figure 3:**
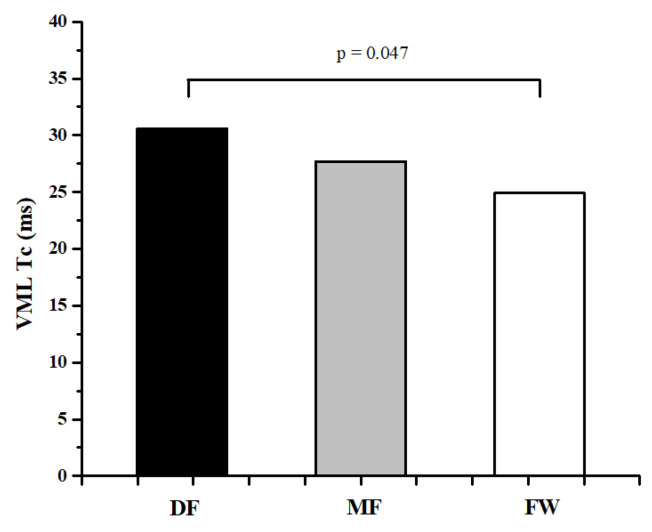
Differences between football players of different playing positions in Tc of VML muscle.

**Figure 4:**
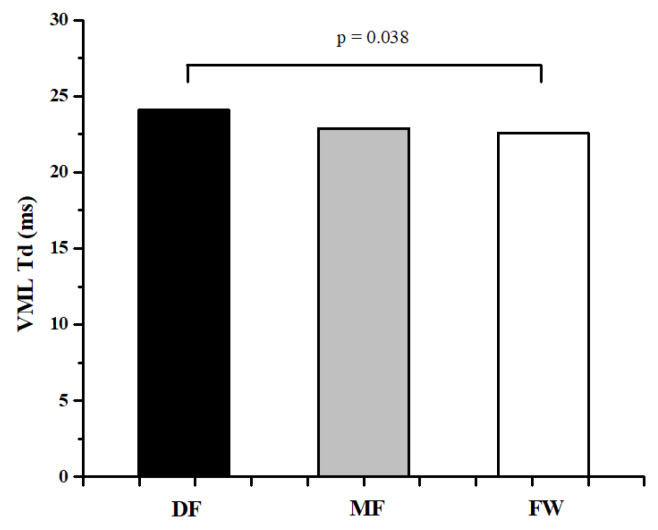
Differences between football players of different playing positions in Td of VML muscle.

**Figure 5:**
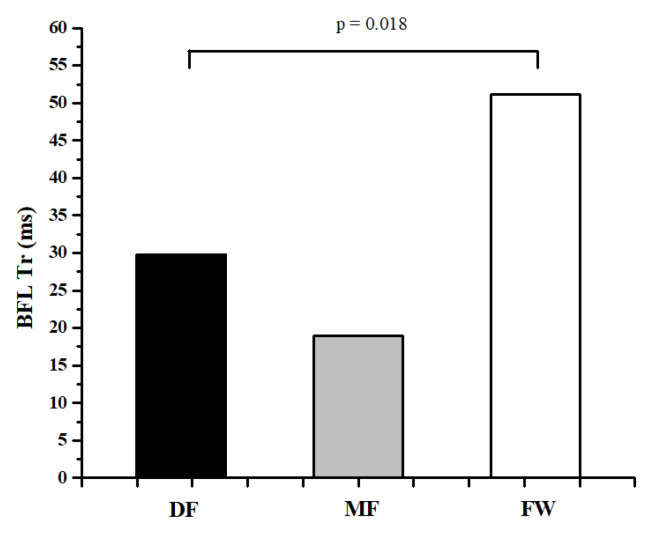
Differences between football players of different playing positions in Tr of BFL muscle.

**Figure 6:**
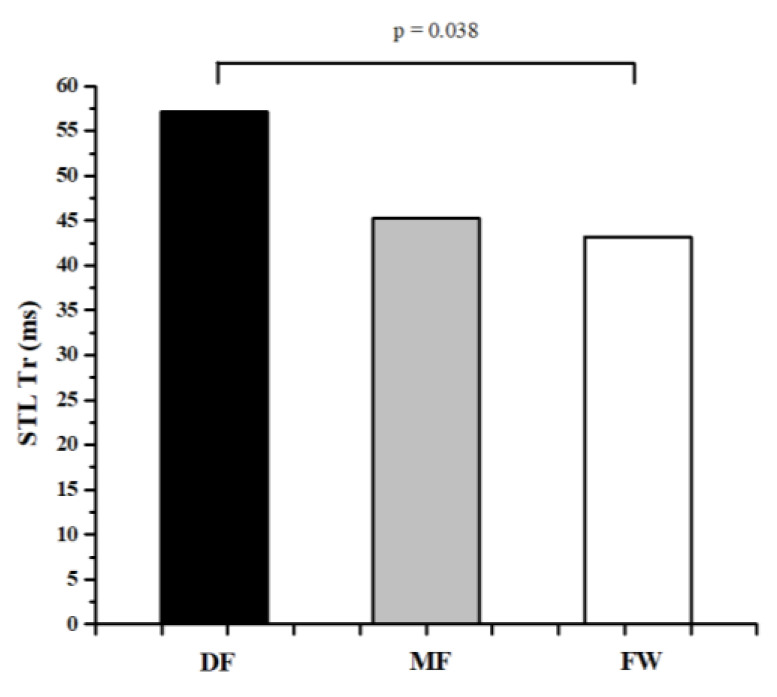
Differences between football players of different playing positions in Tr of STL muscle.

Table 1 and Table 2 represent the descriptive values of measured parameters and results of ANOVA, while Figure 2, Figure 3, Figure 4, Figure 5 and Figure 6 represent the results of the Bonferroni post hoc test in those parameters that showed significant differences between groups. It was noted that only 5 of 50 variables (10%) showed significant differences between groups. Differences in TMG parameters between top-level football players of different playing positions were observed in Tr of RF (F = 4.658, *p* = 0.014) BF (F = 4.433, *p* = 0.016), and ST muscle (F = 3.808, *p* = 0.028), Tc (F = 3.214, *p* = 0.048) and Td (F = 3.705, *p* = 0.031) of the VM muscle. All significant differences were observed in the TMG parameters of the left leg. There were no significant differences between groups in other measured parameters (F = 1.276, *p* = 0.491, on average). The Bonferroni post hoc test results showed that all significant differences appear only between defense and forward players. 

## 4. Discussion

This study investigated the differences in muscle contractile properties measured by the TMG method in top-level football players of different playing positions. The sample consisted of 57 football players from the Serbian Super League. This is the first study that investigated the characteristics of playing positions in football from the aspect of functional and mechanical muscle properties in this sample of participants. It is hypothesized that there will be significant differences in measured parameters between groups.

The results of this study showed that there are small differences in muscle contractile properties measured by the TMG method between football players of different playing positions. Only 5 of 50 variables (10%) showed significant differences between groups (Table 1 and Table 2, Figure 2, Figure 3, Figure 4, Figure 5 and Figure 6). These results are in accordance with other similar studies. The study of Rey et al. [15] showed that differences between football players of different playing positions appear in 3 out of 10 measured TMG parameters (Tc, Tr, and Ts of RF muscle). Another study by Paravlic et al. [16] showed that there are differences in inter-limb symmetry between female football players of different playing positions in only two variables (Tr of VM muscle and Td of GM muscle), while the study of García-García et al. [17] revealed that only the half-relaxation time in the BF and the time to sustain force in the VM differed across all the playing positions considered.

These results can be explained by the specificity of TMG as a method for the assessment of muscle contractile properties, training and selection processes in this group of subjects. Namely, TMG measures functional and mechanical contractile properties which are mainly associated with the ability of the muscle to contract and relax quickly (Tc, Td, Tr), and muscle stiffness (Dm), they do not depend on the subject’s voluntary action and are not associated to a great extent with anthropometric characteristics and body composition, such as body or muscle mass, body fat, etc. Most of the previous findings, regarding differences in muscle contractile properties between football players of different playing positions, show significant differences between these subjects. However, most of these studies focused on muscle strength and power [3,12,14]. As is known, muscle strength and power highly depend on body weight and muscle mass [40], which can differ across playing positions in football [2,4,6]. Also, voluntary activity is an important factor in strength and power testing [41]. Accordingly, football players may differ in various characteristics and abilities, such as anthropometric characteristics, physiological profile, muscle strength, and power, but they do not differ significantly in muscle contractile parameters measured by the TMG method, as it is not influenced by almost any of the named factors; it primarily depends on muscle functional and mechanical characteristics, which are, it can be assumed, similar in football players of all positions due to similar training processes and proper selection processes. 

The obtained results show that differences between football players of different playing positions appear only between DF and FW players (Figure 2, Figure 3, Figure 4, Figure 5 and Figure 6). There are no significant differences in any measured parameters between DF and MF, as well as MF and FW players. DF players had lower Tr of RF (33.9%) and BF muscle (52.6), while higher Tc (20.5%) and Td (6.4%) of VM muscle, and Tr of ST muscle (27.6%), than FW players. Contraction time (Tc), delay time (Td), and relaxation time (Tr) parameters are primarily related to muscle fiber type and muscle fiber fatigue; they represent the twitch force generation speed of muscles, and higher values indicate a low level of muscle contraction velocity, fatigue or the predominance of slow muscle fibers, and vice versa [38,42,43]. Accordingly, it can be concluded that DF players have higher twitch force generation speed and a probable higher percentage of fast twitch muscle fibers when it comes to the main knee flexor and extensor muscles (RF and BF) than FW players, while they have lower contraction velocity and fast twitch muscle fibers when it comes to muscles VM and ST. It can be assumed that these findings are influenced by the tactical, technical, and physiological demands of different playing positions in football. Namely, MF players have several roles in the football team and game tactics (central defensive midfielders, central attacking midfielders, wide midfielders) [44,45,46], they are playing in attacking and defending conditions more than DF and FW players, thus it can be assumed that they are more similar in muscle functional and mechanical properties with players of other positions.

When it comes to measured parameters and muscle groups, it can be concluded that the biggest differences were obtained in the Tr parameter, VM muscle, and left leg. As previously mentioned, the Tr parameter is associated with muscle fiber type, so it can be concluded that the ratio of fast and slow twitch muscle fibers can distinguish DF and FW football players, but not MF players from players of other playing positions. Interestingly, there were no differences in the Dm parameter (Table 1 and Table 2). Maximal displacement (Dm) is associated with muscle stiffness, muscle strength (to some extent), tendon mechanical properties, muscle mass, and fatigue; the decrease in Dm is an indicator of increased muscular stiffness and vice versa [34,35], which implies that playing a different position in football does not influence muscle mechanical properties such as muscle stiffness. One more interesting finding of this study is that all differences were obtained in measured parameters of the left leg, which is in most cases the non-dominant leg (89.4%). This finding is confirming the results of previous studies regarding limb asymmetry in football players, and points to caution since asymmetry in muscle function can lead to injury [16,47,48]. The biggest differences in TMG parameters of VM muscle can point to the fact that VM muscle is most sensitive to detecting differences between football players of different playing positions.

As previously mentioned, the results of this study could provide insight into specific characteristics of playing positions in football, regarding muscle functional and mechanical properties, which could contribute to the development of the training and selection process in football. It can be concluded that the hypothesis, which implies significant differences in muscle contractile properties measured by the TMG method between football players of different playing positions from the Serbian National League, is partially confirmed. The results obtained in this study indicate that the training process in football, from the aspect of functional and mechanical properties such as muscle twitch generation speed and muscle stiffness, should be similar in all players, regardless of playing position. Also, the selection process should be similar for all players. It has been shown that TMG has relatively low sensitive strength for detecting differences between football players of different playing positions. Besides information on playing position characteristics in football players, recommendations for the training and selection process, and the sensitivity of TMG as a method, this study has provided the normative values for TMG parameters of football players from the Serbian Super League, since this is the first study that investigated functional and mechanical muscle properties measured by the TMG method in this large population of top-level football players from this football league. This is also the main limitation of the study since it is focused only on football players from the Serbian Super League.

## 5. Conclusions

This study investigated the differences in muscle contractile properties measured by the TMG method in top-level football players of different playing positions in the sample of players from the Serbian Super League. Study results showed that there are small differences in muscle contractile properties measured by the TMG method between football players of different playing positions. Significant differences were obtained in the Tr of RF, BF, and ST muscle, and the Tc and Td of the VM muscle. All differences were obtained between DF and FW players, and all differences were in the left (non-dominant) leg.

The obtained results showed that the training and selection process in football, from the aspect of functional and mechanical properties, should be similar in all players, regardless of playing position. Also, it has shown that TMG has relatively low sensitive strength for detecting differences between football players of different playing positions. Finally, this study has provided the normative values for TMG parameters of football players from the Serbian Super League.

## Figures and Tables

**Table 1:** Descriptive values (Mean ± SD) and ANOVA results of the TMG parameters of the knee extensor muscles.

		DF	MF	FW	ANOVA	
					F	*p*
RFR	Tc	31.1 ± 6.2	30.4 ± 5	32.3 ± 7.92	0.396	0.675
	Ts	155.6 ± 89.6	132.4 ± 42.4	128.02 ± 37.4	1.118	0.334
	Tr	70.9 ± 29.5	72.7 ± 31.1	71.1 ± 32.4	0.017	0.983
	Dm	6.37 ± 3	6.90 ± 2.45	6.79 ± 2.48	0.210	0.811
	Td	25.2 ± 4.66	23.8 ± 1.91	24.4 ± 2.77	0.737	0.483
RFL	Tc	28.2 ± 5.53	28.8 ± 5.13	28.5 ± 5.25	0.043	0.958
	Ts	103.6 ± 41.2	105.2 ± 42.5	123.6 ± 28.4	1.736	0.186
	Tr	57.3 ± 30.1	58.6 ± 25.6	80.7 ± 24.3	4.658	0.014
	Dm	5.50 ± 2.18	6.43 ± 2.19	6.67 ± 2.1	1.684	0.195
	Td	23.5 ± 2.03	24.2 ± 2.28	24.5 ± 2.7	1.076	0.348
VMR	Tc	28.9 ± 6.3	28.1 ± 6.03	27.4 ± 4.15	0.404	0.669
	Ts	173.3 ± 23.2	177.03 ± 25.6	173.8 ± 19.3	0.132	0.876
	Tr	44.9 ± 18.9	39.8 ± 13.04	41.8 ± 13.2	0.500	0.609
	Dm	6.13 ± 1.68	5.95 ± 1.52	5.48 ± 2.04	0.723	0.490
	Td	22.3 ± 1.46	21.9 ± 1.56	22.07 ± 1.66	0.305	0.738
VML	Tc	30.6 ± 10.1	27.7 ± 6.18	24.9 ± 3.82	3.214	0.048
	Ts	177.4 ± 29.6	178.6 ± 56.8	171.7 ± 47.4	0.128	0.880
	Tr	45.6 ± 17.6	40.2 ± 17.3	48.8 ± 37.2	0.461	0.633
	Dm	5.93 ± 1.78	5.83 ± 1.62	5.42 ± 1.81	0.482	0.620
	Td	24.1 ± 2.24	22.9 ± 1.35	22.6 ± 2	3.705	0.031
VLR	Tc	26.9 ± 5.76	25.4 ± 5.71	24.4 ± 3.67	1.332	0.273
	Ts	123.9 ± 26.4	122.3 ± 34.2	122.2 ± 36.3	0.018	0.982
	Tr	58.06 ± 13.7	57.7 ± 17.2	61.5 ± 27.2	0.211	0.811
	Dm	4.31 ± 1.64	3.97 ± 1.13	3.83 ± 1.33	0.653	0.524
	Td	24.1 ± 3.72	22.8 ± 1.83	22.4 ± 1.39	2.292	0.111
VLL	Tc	25.5 ± 3.83	24.3 ± 4.17	25.1 ± 3.54	0.417	0.661
	Ts	117.5 ± 28.3	123.2 ± 33.9	125 ± 28.1	0.363	0.697
	Tr	65.8 ± 26.5	66.2 ± 27.4	59 ± 15.1	0.581	0.563
	Dm	4.12 ± 1.52	4.38 ± 1.13	4.1 ± 1.45	0.196	0.823
	Td	26.2 ± 12.8	23.7 ± 2.92	22.4 ± 1.86	1.150	0.324

Legend: RF—Rectus Femoris, VM—Vastus Medialis, VL—Vastus Lateralis, R—Right leg, L—Left leg, DF—Defense players, MF—Midfield players, FW—Forward players.

**Table 2:** Descriptive values (Mean ± SD) and ANOVA results of the TMG parameters of the knee flexor muscles.

		DF	MF	FW	ANOVA	
					F	*p*
BFR	Tc	19.5 ± 9.58	26.4 ± 15.3	23.2 ± 11	1.533	0.225
	Ts	132.9 ± 92	144.2 ± 72.4	157.4 ± 84.4	0.441	0.646
	Tr	41.7 ± 35.2	49.3 ± 30.2	41.9 ± 26.6	0.326	0.723
	Dm	1.62 ± 1.41	2.24 ± 1.53	2.77 ± 1.78	2.733	0.074
	Td	24.3 ± 24.5	19.8 ± 5.17	20.5 ± 2.84	0.482	0.620
BFL	Tc	19.8 ± 13.8	18.3 ± 12.1	20.9 ± 12.9	0.193	0.825
	Ts	112.2 ± 70.3	88.7 ± 53.6	132.2 ± 69.3	1.859	0.166
	Tr	29.8 ± 28.9	18.9 ± 20.2	51.1 ± 43.2	4.433	0.016
	Dm	1.31 ± 2.16	1.31 ± 2.23	1.95 ± 2.12	0.569	0.569
	Td	24.05 ± 18,9	19.7 ± 8.31	23.9 ± 17.4	0.371	0.692
STR	Tc	44.9 ± 13.1	46.5 ± 13.1	44.2 ± 13.01	0.139	0.870
	Ts	151.4 ± 54.3	156.6 ± 30.3	147.7 ± 38.1	0.179	0.837
	Tr	64.7 ± 42.7	51.1 ± 19.2	57.1 ± 28.8	0.777	0.465
	Dm	4.99 ± 2.9	5.97 ± 2.51	5.07 ± 2.42	0.694	0.504
	Td	26.08 ± 7.13	25.3 ± 3.78	24.3 ± 4.44	0.508	0.604
STL	Tc	45.7 ± 14.8	46.01 ± 16.3	45.4 ± 14.6	0.005	0.995
	Ts	148.7 ± 35.09	137.3 ± 42.6	139.7 ± 29.1	0.565	0.572
	Tr	57.09 ± 20.1	45.3 ± 16.4	43.2 ± 14.3	3.808	0.028
	Dm	5.99 ± 2.88	5.93 ± 3.14	5.62 ± 2.83	0.093	0.911
	Td	25.9 ± 3.61	25.8 ± 4.58	23.8 ± 3.7	1.884	0.162

Legend: RF—Rectus Femoris, VM—Vastus Medialis, VL—Vastus Lateralis, R—Right leg, L—Left leg, DF—Defense players, MF—Midfield players, FW—Forward players.

## Data Availability

The data are available on request to the corresponding author.
